# Biotechnological production of terpenoids using cell factories

**DOI:** 10.3389/fphar.2025.1621406

**Published:** 2025-10-15

**Authors:** Yalan Si, Shasha Li, Chen Chen

**Affiliations:** 1 Shaanxi Engineering Research Centre for Conservation and Utilization of Botanical Resources, Xi’an Botanical Garden of Shaanxi Province, Institute of Botany of Shaanxi Province, Xi’an, Shaanxi, China; 2 The College of Life Sciences, Northwest University, Xi’an, China

**Keywords:** terpenoids, synthesis, biotechnology, metabolic engineering, enzyme engineering

## Abstract

Terpenoids, as one of the most abundant natural products in nature, have important application values in the fields of medicine, food, and daily chemicals industries. Beyond their industrial applications, terpenoids offer notable nutritional benefits to humans and are extensively utilized for their pharmacological properties. Conventional production approaches, such as plant extraction and chemical synthesis, are increasingly constrained by limited resources and environmental concerns. Consequently, biotechnology has emerged as a pivotal strategy for terpenoid synthesis, owing to its advantages in efficiency, sustainability, and precise regulatory capabilities. This review provides a comprehensive overview of recent progress in biotechnological production of terpenoids, with a particular emphasis on metabolic and enzyme engineering and enzyme engineering. Furthermore, we explored the potential of using computational and artificial intelligence technologies for the rational design and construction of high-performance cell factories is discussed, providing promising pathways for the biosynthesis of terpenoids in the future.

## 1 Introduction

Terpenoids, also known as isoprenoids, are a class of secondary metabolites widely present in nature. Terpenoids are found in almost all unicellular and multicellular organisms and play crucial roles in a variety of biological processes (Tholl, 2015). They can be divided into monoterpenes (C_10_, such as limonene and menthol), sesquiterpenes (C_15_, such as antimalarial drug artemisinin), diterpenes (C_20_, such as anticancer drug paclitaxel), setriterpenes (C_25_, such as anti-inflammatory active substance Manoalide), triterpenes (C_30_, such as immunomodulator ginsenoside), and triterpenes (C_40_, such as pansenoside), and polyterpenes (such as natural rubber) according to the number of carbon atoms (Wang et al., 2021), whose structural diversity determines a wide range of biological activities and applications. In medicine, terpenoids are used in anti-malaria, anti-cancer, anti-inflammatory, and immune regulation. In fragrances and cosmetics, monoterpenes and sesquiterpenes are important components of essential oils and perfumes (Lu et al., 2016; Fuloria et al., 2022). In addition, terpenoids are typically the primary components of essential oils in a majority of plants, contributing a diverse range of aromatic profiles, including floral, fruity, woody, and balsamic notes. Consequently, terpenoids represent a critically important class of compounds within the flavor and fragrance industries (Caputi and Aprea, 2011). Terpenoids are key components of herbivore-induced plant volatiles, which function to deter herbivores or attract their predators. They also act as airborne signals within plants or between neighboring plants. Taking advantage of this characteristic, terpenoid compounds play an important role in agriculture as biocontrol agents (Sharma, et al., 2017).

The biosynthesis of terpenes predominantly occurs through the mevalonate (MVA) and methylerythritol phosphate (MEP) pathways, which generate the universal terpene precursor isopentenyl pyrophosphate (IPP). Subsequently, IPP undergoes diverse modifications, leading to the formation of terpene compounds and their derivatives characterized by varying carbon chain lengths (Li et al., 2023).

**FIGURE 1:**
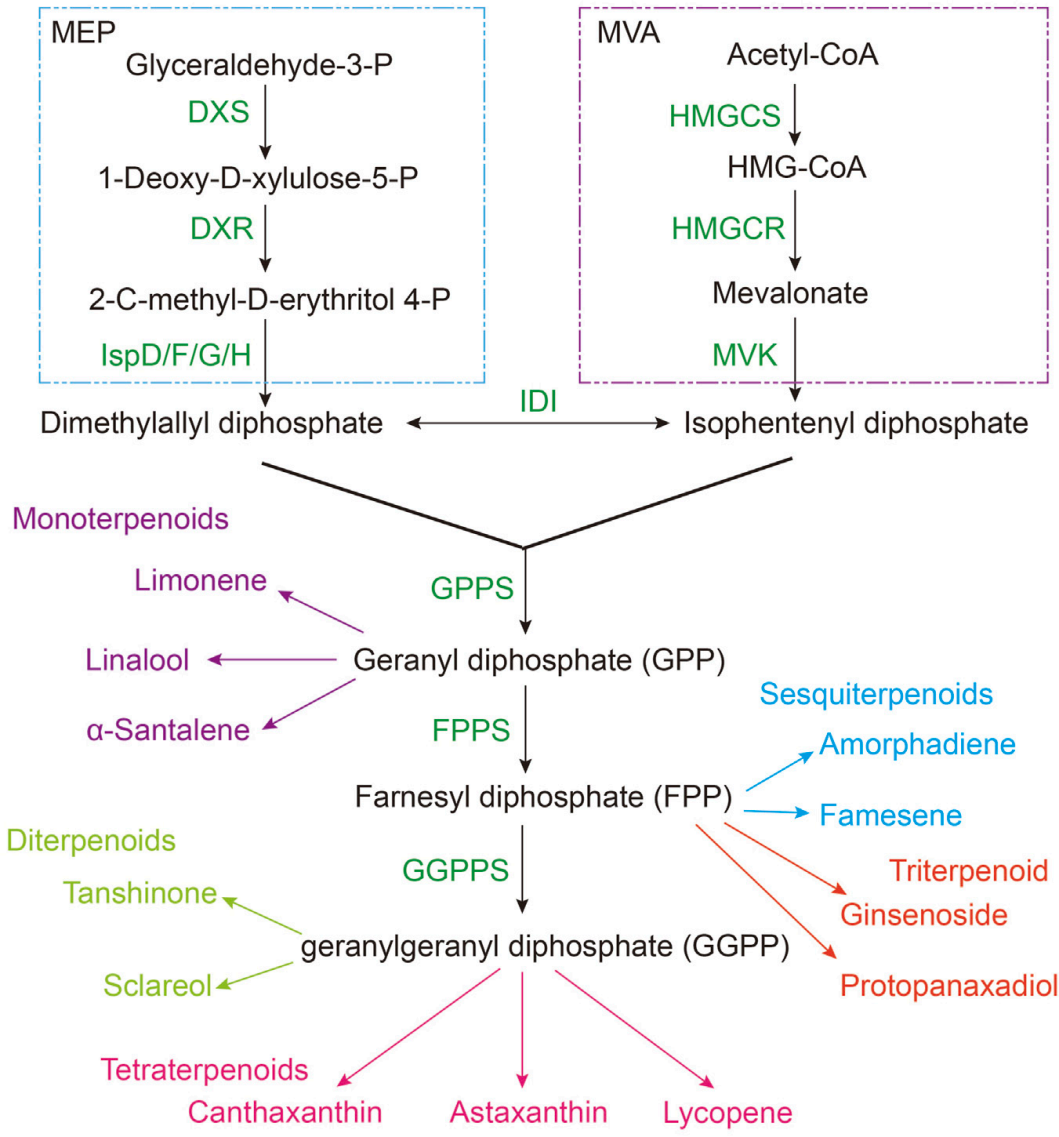
Terpenoids and their common derivatives synthesis by MEP and MVA pathway. MEP, Methylerythritol 4-phosphate pathway; MVA, mevalonate pathway; HMGS, 3-hydroxy-3-methylglutaryl CoA synthase; HMGR, 3-hydroxy-3-methylglutaryl-CoA reductase; HMG-CoA, 3-hydroxy-3-methylglutaryl-CoA; MVK, mevalonate Kinase; IDI, Isopentenyl Diphosphate Isomerase; DXS, 1-deoxyd- xylulose 5-phosphate synthase; DXR, 1-deoxy-d-xylulose 5-phosphate reductoisomerase; FPPS, Farnesyl Pyrophosphate Synthase; GPPS, Geranyl Pyrophosphate Synthase; GGPPS, Geranylgeranyl Pyrophosphate Synthase.

The mevalonate (MVA) pathway, extensively conserved among eukaryotes—including animals and fungi—as well as certain archaea, utilizes acetyl-CoA as the initial substrate to produce isopentenyl pyrophosphate (IPP) and its isomer dimethylallyl pyrophosphate (DMAPP) through a series of enzymatic reactions, which ultimately participate in the synthesis of chlorophyll, carotenoids, ubiquinones, and plant hormones such as gibberellin, brassinosteroids (Figure 1). Initially, acetyl-CoA is converted into acetoacetyl-CoA by the enzyme acetoacetyl-CoA thiolase (AACT). Subsequently, 3-hydroxy-3-methylglutaryl-CoA synthase (HMGS) catalyzes the formation of 3-hydroxy-3-methylglutaryl-CoA (HMG-CoA) from acetyl-CoA. HMG-CoA is then reduced to mevalonate (MVA) via the action of 3-hydroxy-3-methylglutaryl-CoA reductase (HMGCR). Mevalonate undergoes phosphorylation by mevalonate kinase (MVK) and phosphomevalonate kinase (PMK), among other enzymes, resulting in the production of mevalonate phosphate (MVAP), mevalonate diphosphate (MVAPP), and ultimately IPP and DMAPP. These two isoprenoid intermediates represent common end products of both the MVA and alternative pathways, which subsequently participate in the enzymatic synthesis of diverse terpenoid compounds. While the methylerythritol phosphate (MEP) pathway is predominantly present in prokaryotic organisms, including most bacteria and cyanobacteria, as well as in plant plastids. This biosynthetic route initiates with pyruvate and glyceraldehyde-3-phosphate (G3P) and proceeds through a series of enzymatic reactions to yield isopentenyl pyrophosphate (IPP) and dimethylallyl pyrophosphate (DMAPP). These intermediates serve as precursors for the biosynthesis of various terpenoid compounds. For instance, geranylgeranyl pyrophosphate (GGPP) is synthesized via the action of geranylgeranyl pyrophosphate synthase (GGPS) and functions as a substrate for diterpene production. Additionally, farnesyl pyrophosphate (FPP), generated by farnesyl pyrophosphate synthase (FPPS), is subsequently converted into squalene by squalene synthase (SQS), which can then be further metabolized into sterols (Kumari et al., 2024).

## 2 Metabolic engineering strategies

With the identification of an increasing number of critical enzymes involved in terpenoids biosynthesis pathways, the elucidation of these pathways has progressively advanced. Researchers have endeavored to achieve *in vitro* terpene synthesis within host organisms such as *Escherichia coli* and yeast, attaining notable breakthroughs in the production of pharmaceutical compounds, fragrances, and industrial raw materials. Their investigative approaches primarily concentrate on the selection of appropriate host cells, the modulation of enzyme gene expression levels, and the structural optimization of enzymes.

### 2.1 Host cells

By transferring the target metabolic pathways to optimized host systems such as *E. coli*, yeast, filamentous fungi, and plant chassis, combined with metabolic engineering strategies, heterologous biosynthesis of terpenoids has significantly improved product diversity and synthesis efficiency. The traditional production model is limited by the complex metabolic regulation of the natural host, the difficulty of culture, and the low transformation efficiency, prompting researchers to transfer the key gene clusters to heterologous systems with excellent developmental characteristics for reconstruction. Prokaryotic hosts, such as *E. coli*, have become the preferred platform for terpene biosynthesis due to their rapid proliferation ability, clear metabolic background, and mature gene manipulation system. It has successfully achieved efficient synthesis of high-value products such as *β-farnesene* (1.3 g/L) by enhancing the supply of isoprene precursors (IPP/DMAPP) (Xu et al., 2018).

Although photosynthetic cyanobacteria exhibit limited carbon fixation capacity, their endogenous isoprenoid metabolic flux is remains inadequate, which limits the efficiency of terpenoid production. It is necessary to overexpress terpene synthase and key enzymes of the MEP pathway to improve the production of terpenoids. Notably, marine diatoms have demonstrated successfully expression of plant-derived metabolites, such as anti-HIV terpenoid botulinum toxin, facilitated by their simplified cellular architecture and well-established genetic manipulation platform (Ben David et al., 2022). Furthermore, the eukaryotic host yeast shows unique systemic advantages: its natural MVA pathway provides a stable metabolic framework for terpenoid precursor synthesis, while its endoplasmic reticulum machinery efficiently facilitates the proper folding of plant-derived cytochrome P450 and UbiA transmembrane proteins. These features have enabled the successful heterologous production of complex molecules such as ginsenosides.

Despite significant advancements in microbial chassis systems, challenges persist, including the accumulation of toxic intermediates and metabolic network imbalances. Therefore, *in vitro* culture systems-such as plant cell suspension culture and hairy root cultures-have increasingly emerged as vital complementary approaches for synthesizing complex terpenoids (Grover et al., 2012; Lacchini et al., 2025). These systems leverage inherent metabolic compartmentalization and possess substantial potential for large-scale production. By emulating the endogenous regulatory environment of plants, they effectively circumvent common metabolic disruptions encountered in microbial hosts, thereby offering novel strategies for the industrial-scale biosynthesis of high-value terpenoids.

### 2.2 Elevated expression levels of critical enzymes

Plant gene engineering offers a potent approach to enhance the biosynthetic flux of secondary metabolites by specifically targeting the key rate-limiting enzymes in biosynthetic pathways and their metabolic network nodes. Central to this technology is the integration of multifaceted regulatory strategies. Firstly, through analyses of subcellular compartmentalization, gene expression network modeling, and epigenetic regulatory mechanisms, a heterologous overexpression system for rate-limiting enzymes was constructed to remove the metabolic bottlenecks. Secondly, tissue-specific promoter elements were used to achieve spatially precise enrichment of target metabolites. Meanwhile, the distribution of precursor metabolic flow was dynamically optimized by the heterologous co-expression system of key enzyme genes in MEP and MVA pathways. This strategy overcomes the dual limitations of insufficient precursor availability and compartmentalized product accumulation characteristic of conventional metabolic engineering approaches, thereby establishing a precise regulatory framework for plant synthetic biology. In the field of terpenoid metabolic engineering, several breakthrough studies have verified the effectiveness of synthetic biology strategies. Based on the heterologous expression system mediated by *Agrobacterium tumefaciens*, stable expression of geraniol synthetases in tobacco hairy roots, and successfully catalyzed the production of six structurally diverse glycosylation geraniol derivatives and revealing the metabolic plasticity of the hair root system in terpenoid glycosylation (Chang et al., 2025). Furthermore, constitutive overexpression of the lavender-derived terpenoid synthase gene LIS, coupled with optimization of plastidic transport peptide subcellular localization, significantly enhanced linalool synthesis throughput, presenting a novel strategy for large-scale production of monoterpene fragrance compounds (Mendoza-Poudereux et al., 2014). Additionally, directed evolution techniques applied to the functional domain of artemisinin sesquiterpene cyclase surmounted catalytic efficiency limitations, resulting in a 30% increase in artemisinin biosynthesis and establishing a paradigm for key enzyme optimization in antimalarial drug metabolic engineering (Mirzaee et al., 2016).

Despite these advances, the total biosynthesis of complex metabolites remains challenged by factors such as multi-gene cooperative regulation, feedback inhibition, and metabolic flux allocation. Consequently, optimizing flux balance through dynamic regulatory systems or transient expression via viral vectors is imperative. The field has progressed from single-gene manipulations toward comprehensive multi-network regulatory frameworks. Looking forward, these advancements are poised to facilitate the sustainable production of nutritionally enhanced crops, anticancer therapeutics, and high-value fragrance compounds, while providing innovative models for the design of plant chassis in synthetic biology applications. Beyond the overexpression of key enzyme-encoding genes within the terpene biosynthesis pathway, metabolic flux can be modulated through gene knockout or knockdown strategies. For instance, genome editing technologies based on the CRISPR-Cas9 system offer a precise and efficient platform for genetic manipulation in plant metabolic engineering (Das et al., 2024). This approach enables stable and heritable reconstruction of metabolic networks via coordinated regulation of multiple genes. Owing to its high editing efficiency, low off-target risk, and broad species compatibility, including both prokaryotic and eukaryotic systems, CRISPR-Cas9 has been effectively applied for multidimensional regulation of key nodes in metabolic pathways. Empirical evidence demonstrates that knockout of *LCY-B1/B2/CYC-E* in tomato, which blocks the conversion of lycopene to carotene, results in a 5-fold increase in lycopene content by enhancing the expression of beneficial genes through suppression of competing pathways (Li et al., 2018). Similarly, editing the tanshinone synthase gene in *Salvia miltiorrhiza* inhibits the competing phenolic metabolism and redirecting the metabolic flow to terpenoids, achieving a 30% increase in tanshinone production (Li et al., 2017).

In recent years, RNA interference (RNAi) technology has also advanced significantly in the context of terpenoid biosynthesis, improving biosynthetic efficiency and expanding metabolic diversity through precise regulation of key pathway genes. For example, Fu et al. (2017) reported that silencing In *Artemisia annua*, the *AaPDR3* gene in *A. annua* led to a reduction in β-caryophylene levels and an approximately 20% increase in artemisinin synthesis. Additional studies have made notable contributions, such as the silencing of limonene synthase in spearmint and the heterologous expression of geraniol and linalool synthase genes, which substantially enhanced the production of monoterpene derivatives, particularly geraniol (Li et al., 2020).

### 2.3 Enzyme engineering optimization

The biosynthesis of terpenoids is fundamentally reliant on enzyme-catalyzed reaction cascades, characterized by a complex pathway comprising multiple steps catalyzed by key enzymes. Enzyme engineering has emerged as a pivotal technology for enhancing both the yield and structural diversity of terpenoids through the modification of enzyme activity, stability, substrate specificity, and regulatory functions. The optimization of enzyme engineering strategies represents a central approach to augmenting terpenoid production. Terpenoid biosynthesis can be divided into three stages: upstream (precursor supply), midstream (skeleton construction), and downstream (structural modification). A summary of the enzymes involved in the terpenoid synthesis pathway is shown in Table 1 below.

**TABLE 1:** Summary of strategies for microbial terpenoid production.

Terpenoids and derivatives	Strategies	Enzyme/gene	Organism	Highest reported titre	References
Linalool	Overexpression of MVA pathway in both mitochondria and cytoplasm, and downregulation of ERG20 and overexpressing tHMG1 improved linalool production	farnesyl pyrophosphate synthase, tHMG1	*Saccharomyces cerevisiae*	23.45 mg/L in a 2 L fermenter	Zhang et al. (2020)
α-santalene	Reconstruct the whole MVA pathway in mitochondria to harness the precursor pools	COX4	*Saccharomyces cerevisiae*	41 mg/L	Dong et al. (2021)
Amorphadiene	A push-and-pull strategy in overexpressing HMG-CoA and acetyl-CoAby Gene, either by inhibiting fatty acids synthase or activating the fatty acid degradation pathway	HMG-CoA, acetyl-CoA	*Yarrowia lipolytica*	171.5 mg/L	Marsafari and Xu (2020)
Ginsenoside	engineered yeast for complex natural product production by targeting the ER-localized PPDS to LDs (via PLN1) to enhance DD-to-PPD conversion, reconstituting the CK biosynthetic pathway in the PPD-producing chassis, and boosting CK production by upregulating the Pn3-29 gene module, ultimately achieving high CK titers in fed-batch fermentation	cytochrome P450 enzyme protopanaxadiol synthase (PPDS)	*Saccharomyces cerevisiae*	5 g/L	Shi et al. (2021)
Protopanaxadiol	Construction of the protopanaxadiol pathway in peroxisome	Pex11p, Pex34p, and Atg36p	*Saccharomyces cerevisiae*	4.1 mg/L	Choi et al. (2022)
Lycopene	Three mutation sites (V13D, S148I, and V301E) were introduced to enhance lycopene production, with V13D located near the MK catalytic center	mevalonate kinase	*Saccharomyces cerevisiae*	A lycopene production of 1.43 g/L	Chen et al. (2018)
Canthaxanthin	Introduce the beita-carotene ketolase variant OBKTM29 to the plasma membrane and overexpression the *Pdr1* and *Pdr3* to improve the stress tolerance	drug resistance (PDR) regulators *Pdr1* and *Pdr3*	*Saccharomyces cerevisiae*	1.44 g/L	Chen et al. (2022)
Astaxanthin	Fusing β-carotene ketolase and hydroxylase, and targeting the astaxanthin pathway to subcellular organelles individually not only accelerated the conversion of β-carotene to astaxanthin but also significantly reduced the accumulation of ketocarotenoid intermediates	β-carotene ketolase, hydroxylase	*Yarrowia lipolytica*	858 mg/L	Ma et al. (2021)
Four terpenoids	EcoMine strategy, we identified and assembled a translationally coupled TriMEP cassette containing key rate-limiting MEP pathway genes from a high-producing strain and expressed it under strong constitutive promoters		*Streptomyces*	(−)-*epi*-α-bisabolol (1, 237 ± 14 mg/L), *ent*-(13*Z*)-isocupressic acid (2, 173 ± 14 mg/L), *ent*-(13*E*)-isocupressic acid (3, 209 ± 20 mg/L), and *ent*-atiserenoic acid (4, 325 ± 17 mg/L)	Lin et al. (2025)

The intermediate phase pertains to the assembly of the terpenoid backbone, which can be optimized through rational design of enzyme active sites, such as mutagenesis of substrate-binding pocket residues, or through enzyme domain fusion strategies, exemplified by the fusion of terpene synthases (TPS) with isopentenyl transferase (Zhou et al., 2024). The downstream stage encompasses structural modification and functionalization of terpenoids, predominantly mediated by cytochrome P450 enzymes. Cytochrome P450 enzymes play a leading role in the oxidative transformation of terpenoids, participating in over 97% of such modifications (Xiao et al., 2019), thereby serving as essential molecular tools for generating the extensive structural and functional diversity observed in terpenoids. Characterized by a heme thiol prosthetic group, cytochrome P450 enzymes form a distinctive reduced carbon monoxide complex exhibiting an absorption peak at 450 nm in the UV-VIS spectrum, which underpins their systematic nomenclature (Mak and Denisov, 2018; Hu et al., 2023). Phylogenetic analysis shows that P450 enzymes are widely distributed and show a high degree of evolutionary conservation and functional plasticity (Crešnar et al., 2011). The catalytic activity of P450 enzymes depends on a specialized redox chaperone system that facilitates the activation of molecular oxygen into highly reactive intermediates through continuous electron transfer. This unique mechanism enables precise, regioselective, and stereoselective insertion of oxygen atoms into inert C-H bonds, yielding diverse oxidative modifications such as hydroxylation and epoxidation. Consequently, this catalytic property not only enhances the structural complexity of terpenoids but also underlies the chemical basis for their broad spectrum of biological activities.

Enhancement of local enzyme concentration represents a primary strategy to improve P450 function. In the terpenoid biosynthesis system of *Saccharomyces cerevisiae*, the inefficient expression of cytochrome P450 enzymes is often limited by the insufficient local concentration of enzyme molecules due to their subcellular localization (Jiang et al., 2021). To address this bottleneck, researchers have developed multi-dimensional engineering strategies to optimize P450 expression performance: Gene expression system enhancement, which significantly enhances P450 transcript levels by promoter strength adjustment (e.g., replacement of the strong constitutive promoter *ADH1* or inducible promoter *GAL1*) and gene dosage optimization (multicopy integration or episomal plasmid amplification) (Hu et al., 2023). In addition, engineering of the N-terminal sequence, such as truncation of the hydrophobic transmembrane domain or fusion with secretory signal peptides, have been employed to improve folding efficiency and soluble expression of heterologous P450 in yeast. Subcellular compartmentalization strategies focus on the endoplasmic reticulum (ER), the principal site of eukaryotic P450 activity, by engineering a spatially confined catalytic microenvironment. This involves overexpression of ER-resident proteins, such as protein disulfide isomerase (PDI) and chaperones, such as *Kar2/BiP* to facilitate cotranslational folding and stabilize P450 enzymes (Huang et al., 2018). Metabolic channel remodeling further co-localizes P450 with terpenoid precursor synthetases within ER membrane microdomain, thereby reducing substrate diffusion distances and enhancing cascade reaction kinetics (Jin et al., 2022). Systems biology and integrative omics analyses have elucidated that P450 functionality is depended on the simultaneous enhancement of NADPH regeneration system and heme prosthetic biosynthesis network (Liu et al., 2025). Global regulation of heme synthesis pathway and REDOX partners, P450 catalytic activity can be systematically enhanced. Together, these strategies demonstrate that compartmentalization-based metabolic engineering strategies not only break the bottleneck of P450 expression but also provide a scalable chassis optimization paradigms for efficient biological manufacturing of complex terpenoids.

Improvement of catalytic performance constitutes another critical avenue for enhancing P450 enzyme utility. In *S. cerevisiae* terpenoid biosynthesis system, catalytic activity and substrate specificity of cytochrome P450 enzymes are the key determinants of synthetic yield and product purity (Kim et al., 2022). To address these parameters, multifaceted engineering strategies have been established. The catalytic efficiency of P450 enzymes is tightly dependent on the electron transport chain mediated by REDOX chaperone systems such as cytochrome P450 reductase (CPR), which involves a cascade of electron transfer from NADPH→FAD→FMN→ heme iron ions (Cheng et al., 2024). Strategies to enhance catalytic activity include optimization of cofactor availablityvia overexpression of NADPH-regeneration enzymes such as glucose-6-phosphate dehydrogenase (G6PDH) and augmentation of the heme biosynthesis pathway. Co-localization of REDOX partners is achieved through gene fusion or subcellular co-targeting techniques to construct P450-CPR complexes, thereby minimizing electron transfer distances. Regulation of heme homeostasis, through introduction of exogenous heme transport systems or inhibition of heme-degrading enzymes, preserves the integrity of the enzyme’s active site. Furthermore, advanced protein engineering approaches aimed at modifying substrate channels and binding pockets enhance substrate specificity, thereby improving the chemical purity of target terpenoids. Conversely, the discovery of new terpenoids is facilitated by mining and engineering P450 enzymes with different substrate specificities, expanding the repertoire of accessible terpenoid structres (Wang et al., 2024).

### 2.4 Alternative synthetic approaches

#### 2.4.1 Engineering terpenoid biosynthetic pathways within distinct cellular organelles

Numerous successful instances have been reported in the construction of heterologous pathways for terpenoid biosynthesis within yeast, an organism characterized by a diverse array of organelles and membrane structures, including endoplasmic reticulum, peroxisomes, mitochondria, and lipid droplets. The precise targeting of each engineering pathway or its components to the appropriate organelle is imperative, given that each organelle offers a unique physicochemical environment and a unique composition of metabolites, enzymes, and cofactors.

The endoplasmic reticulum (ER), recognized as the quality control hub for protein folding in eukaryotic cells, employs a sophisticated multi-level regulatory network to meticulously govern the synthesis, folding, post-translational modification, subcellular localization and functional expression of P450 enzymes (Brignac-Huber et al., 2016). Its central role is to ensure the three-dimensional conformational integrity of newly synthesized P450 enzymes through the chaperone system. Additionally, coordinate post-translational modifications, such as glycosylation, to form the electron transport system required for the active center. At present, ER-targeted engineering mainly focuses on microenvironment optimization strategies; for instance, overexpression of INO2 or knockdown of OPI1, a regulator of phospholipid metabolism, significantly expands the ER membrane surface area, thereby fostering the formation of high-density functional domains of P450 enzymes functional domains (Grogan, 2021). For example, in the synthesis of 11, 20-dihydroxyiron glycol, the combined application of ER remodeling and REDOX balance regulation strategies has yield a 42.1-fold increase in product yield, underscoring the amplification effect of ER microenvironment modulation on P450 catalytic activity (Cheng et al., 2024).

Mitochondria, as the center organelles of cellular energy metabolism, provide an ideal environment for terpenoid biosynthesis due to their unique physiological characteristics. TCA cycle continuously outputs high concentrations of acetyl-CoA, directly supplying precursors for the MVA pathway (Yuan et al., 2016). Furthermore, the inner mitochondrial membrane acts as a physical barrier that restricts the diffusion of intermediates, minimizing the diversion of metabolic flux into competing pathways. The ATP-enriched environment drives efficient catalysis of terpenoid synthase. Through the precise positioning strategy, the key enzyme system of the MVA pathway in the mitochondria of *S. cerevisiae* and a geraniol synthesis module, which successfully achieved compartmental synthesis. This approach not only increased geraniol production 6 times compared to the cytoplasmic system but also displayed a unique energy coupling advantage by harnessing the mitochondrial proton gradient to drive transmembrane transport of terpene precursors (Yee et al., 2019).

#### 2.4.2 High-throughput screening utilizing automated workstations

The conventional approach of analyzing individual genes or gene clusters encounters three principal challenges: the complexity in predicting products due to the overlapping substrate specificity of terpene synthases (TS); limited expression compatibility of secondary metabolite biosynthetic gene clusters (BGCs); and an imbalance in metabolic flux between chemical structure elucidation and bioactivity assessment. Collectively, these factors have resulted in a prolonged stagnation in terpenoid discovery efficiency, which remains low at approximately 1.2–3.5 new compounds/kilogene cluster (Wei et al., 2021). Nowadays, to bridge the gap between selectively extracting biosynthetic gene clusters (BGCs) for untapped natural products and the abundance of available genome sequences, a fungal genome mining tool was developed (Tang and Matsuda, 2024). This tool identifies BGCs encoding enzymes that lack detectable protein domains (i.e., domainless enzymes) and are not recognized as biosynthetic proteins by existing bioinformatic methods. For example, BGCs encoding homologues of Pyr4-family terpene cyclase were searched in approximately 2,000 fungal genomes, leading to the discovery of several BGCs with unique features.

## 3 Future and prospects

At present, traditional microorganisms are the main hosts for terpenoids synthesis, but their metabolic flux, substrate tolerance and product storage capacity for terpenoids accumulation remain constrained. Future research should prioritize the exploration and development of non-traditional host organisms, including extremophilic microorganisms such as halophilic and acidophilic strains. These organisms possess the ability to thrive under harsh industrial fermentation conditions, thereby minimizing contamination risks. Additionally, plant cells and fungi represent promising platforms due to the presence of unique terpene synthases (TPS) and terpene cyclases (TTC) identified in non-model plant species, which offer novel components for heterologous expression systems. Filamentous fungi, exemplified by A. termitophilus, have demonstrated the capacity for elevated triterpene production through medium optimization, indicating their potential for industrial-scale applications. Furthermore, advances in synthetic biology enable the construction of “superhosts” by integrating key metabolic modules from multiple species, resulting in chimeric hosts characterized by enhanced precursor supply and dynamic regulatory capabilities.

Current metabolic engineering strategies relies on static approaches, such as constitutive overexpression or gene knockout. Moving forward, it is imperative to integrate multidimensional optimization approaches that incorporate dynamic regulatory systems. These systems utilize transcription factors or metabolic sensors to regulate gene expression in real time, balancing between cell growth and metabolites synthesis. For example, the dynamic regulation of acetyl-CoA synthesis through terpenoid precursor flux can be achieved via PciF1-mediated REDOX state sensing. Furthermore, subcellular compartmentalization and pathway partitioning represent effective strategies to enhance metabolic efficiency. Targeting the terpenoid synthesis pathway to peroxisomes or lipid droplets minimizes substrate competition and enhances product accumulation. Notably, mitochondrial compartmentalization has been demonstrated to increase β-bisabolene production by 5-fold (Iovine et al., 2021). In addition, the development of green bioprocesses and alternative carbon sources is critical for sustainable production. This includes the utilization of substrates such as xylose, methanol, or industrial waste streams, alongside the integration of gene modules enabling the metabolism of non-food-grade carbon sources. Finally, the application of high-throughput screening combined with machine learning-assisted design offers a powerful approach to optimize metabolic pathways. By constructing predictive models based on transcriptomic and metabolomic datasets, it is possible to rapidly identify efficient enzyme variants or optimize ribosome binding site sequences to achieve balanced metabolic flux.

The chemical diversity of natural terpenoids remains incompletely explored. Advancing this field will require the integration of computational biology and synthetic biology approaches to predict novel structures and functions. Artificial intelligence-based enzyme function prediction, employing tools such as AlphaFold, can facilitate the elucidation of catalytic mechanisms in terpenoid synthases from non-model organisms, enabling the prediction of substrate specificity and product outcomes. Additionally, reverse engineering of biosynthetic pathways through the construction of virtual metabolic networks can inform the heterologous assembly of pathways, guided by the underlying chemical logic of the terpenoid backbone. Furthermore, the rational design of unnatural terpenoids can be achieved by synthesizing bioactive compounds with non-natural modifications via modular enzyme engineering or chemo-enzymatic tandem reactions.
