# Correction: Biotechnological production of terpenoids using cell factories

**DOI:** 10.3389/fphar.2025.1729872

**Published:** 2025-11-10

**Authors:** Yalan Si, Shasha Li, Chen Chen

**Affiliations:** 1 Shaanxi Engineering Research Centre for Conservation and Utilization of Botanical Resources, Xi’an Botanical Garden of Shaanxi Province, Institute of Botany of Shaanxi Province, Xi’an, Shaanxi, China; 2 The College of Life Sciences, Northwest University, Xi’an, China

**Keywords:** terpenoids, synthesis, biotechnology, metabolic engineering, enzyme engineering

Affiliations “Shaanxi Engineering Research Centre for Conservation and Utilization of Botanical Resources, Xi’an Botanical Garden of Shaanxi Province, Institute of Botany of Shaanxi Province, Xi’an, Shaanxi, China” and “The College of Life Sciences, Northwest University, Xi’an, China” were published in the wrong order. Their positions have been swapped. **Affiliation 1** is now “Shaanxi Engineering Research Centre for Conservation and Utilization of Botanical Resources, Xi’an Botanical Garden of Shaanxi Province, Institute of Botany of Shaanxi Province, Xi’an, Shaanxi, China”, while **Affiliation 2** is now “The College of Life Sciences, Northwest University, Xi’an, China” and **Affiliation 2** were swapped. Authors Shasha Li and Chen Chen are now assigned to **Affiliation 1**.

Yalan Si was previously only affiliated with **Affiliation 2** “The College of Life Sciences, Northwest University, Xi’an, China”. **Affiliation 1** “Shaanxi Engineering Research Centre for Conservation and Utilization of Botanical Resources, Xi’an Botanical Garden of Shaanxi Province, Institute of Botany of Shaanxi Province, Xi’an, Shaanxi, China”, was omitted for author Yalan Si. This affiliation has now been added for the author.

The original article has been updated.

